# Disseminated peritoneal coccidioidomycosis mimicking malignancy in an immunosuppressed patient: a case report

**DOI:** 10.1093/jscr/rjaf978

**Published:** 2025-12-12

**Authors:** Ryan Meyer, Tamir Bresler, Kevin Palmer, Makrouhi Kademian

**Affiliations:** Department of Surgery, Los Robles Regional Medical Center, 215 W Janss Rd, Thousand Oaks, CA 91360, United States; Department of Surgery, Los Robles Regional Medical Center, 215 W Janss Rd, Thousand Oaks, CA 91360, United States; Department of Surgery, Los Robles Regional Medical Center, 215 W Janss Rd, Thousand Oaks, CA 91360, United States; Department of Surgery, Los Robles Regional Medical Center, 215 W Janss Rd, Thousand Oaks, CA 91360, United States

**Keywords:** disseminated coccidioidomycosis, ustekinumab, peritoneal coccidioidomycosis

## Abstract

Disseminated peritoneal coccidioidomycosis is rare and can mimic peritoneal carcinomatosis. Ustekinumab, an IL-12/23 inhibitor, impairs Th1/Th17 immunity which is critical for fungal control. No prior cases link ustekinumab use to disseminated fungal disease. We report a 45-year-old woman from an endemic region with prior pulmonary coccidioidomycosis and psoriatic arthritis on ustekinumab who presented with malignant-appearing ascites and omental caking. Fluid studies were non-diagnostic, but laparoscopic biopsies confirmed disseminated Coccidioides infection. She improved with liposomal amphotericin B followed by oral fluconazole suppression. This case highlights the need to consider peritoneal coccidioidomycosis in immunosuppressed patients, particularly on IL-12/23 blockade, and underscores the role of tissue diagnosis when fluid studies are negative.

## Introduction

Coccidioides spp. cause ~150 000 infections annually in the United States, with an estimated 1% progressing to disseminated disease, which includes a substantial increase in mortality risk [1, 2]. The most common extrapulmonary sites are skin, bones, and the central nervous system. Peritoneal involvement is uncommon and largely described in isolated case reports or small series [3, 4]. Clinically, peritoneal coccidioidomycosis often resembles peritoneal carcinomatosis or tuberculous peritonitis, which can delay evaluation and appropriate treatment [3]. Immunosuppression is a recognized risk for dissemination [5]. Ustekinumab has a therapeutic effect on interleukins that are essential for antifungal immunity [6, 7]. This case study reports a rare case of peritoneal coccidioidomycosis in a patient on ustekinumab.

## Case report

A 45-year-old woman living in California presented with 6 weeks of abdominal pain associated with progressive distension, nausea, early satiety, and dyspnea. Past history included pulmonary coccidioidomycosis previously treated with fluconazole and psoriatic arthritis controlled with ustekinumab. She had discontinued antifungal therapy ~2 months prior to presentation. On examination, she had abdominal distension with mild diffuse tenderness.

**Figure 1:**
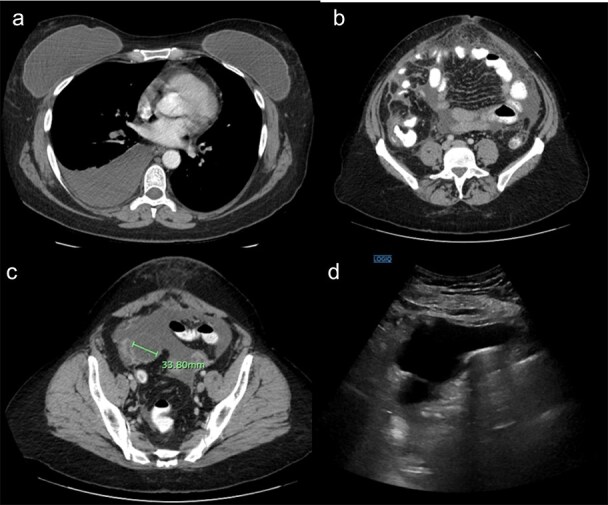
(a) Demonstrating right pleural effusion, likely from pulmonary coccidioidomycosis. (b) Demonstrating thickened anterior omental fat concerning for peritoneal carcinomatosis. (c) Demonstrating a right adnexal mass. (d) Ultrasound demonstrating a large amount of ascites.

**Figure 2:**
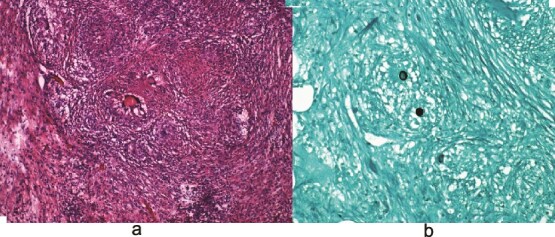
(a) Hematoxylin and eosin (H&E) images demonstrating granulomas. (b) Grocott-Gomori methenamine silver (GMS) stain highlighting fungal organisms.

Laboratory testing revealed leukocytosis (WBC 11.7 × 10^9^/L) and elevated CA-125 (516 U/mL). Contrast-enhanced computed tomagraphy (CT) demonstrated moderate ascites, omental caking, nodular peritoneal thickening, a small adnexal mass, and bilateral pleural effusions, (Fig. 1). These features were concerning for an advanced gynecologic malignancy. Diagnostic paracentesis and thoracentesis were non-diagnostic: cytology was negative for malignant cells and no organisms were identified on routine stains or cultures. Given that there was persistent concern for carcinomatosis, she underwent diagnostic laparoscopy. Intraoperatively there was diffuse peritoneal studding and straw-colored ascites. Biopsies revealed necrotizing granulomatous inflammation containing thick-walled spherules consistent with Coccidioides with fungal cultures subsequently confirming the diagnosis (Fig. 2).

She was treated with IV amphotericin B initially due to extensive peritoneal involvement and symptom burden, then transitioned to oral fluconazole for long-term suppression. Ustekinumab was held during the acute period. At 6-month follow-up, she reported improved functional status with decreasing ascites and no radiographic evidence of carcinomatosis. She remained on azole therapy under Infectious Disease supervision.

## Discussion

### Epidemiology and differential diagnosis

Disseminated disease occurs in roughly 1% of coccidioidal infections and carries meaningful morbidity and mortality of up to 20%–30% even in patients receiving appropriate treatment. [1, 2] Peritoneal involvement is rare. Case reports and series show that patients with peritoneal involvement are often first worked up for malignancy or other causes of ascites prior to the diagnosis being made. [3, 4]. Elevations in tumor markers, such as CA-125 may further mislead clinicians toward a malignant diagnosis.

### Why ustekinumab matters: Immunology and clinical signal

IL-12 promotes Th1 differentiation and IFN-γ production, crucial for macrophage activation against intracellular fungi; IL-23 supports Th17-mediated mucosal and neutrophil responses [6]. In murine models, administering IL-12 decreases fungal burden after intraperitoneal Coccidioides challenge, whereas IL-12 neutralization increases pulmonary and extrapulmonary loads [6]. Humans with defects in the IL-12/IFN-γ axis develop severe disseminated coccidioidomycosis, underscoring pathway importance [6, 7]. Ustekinumab’s p40 blockade is therefore plausibly a risk factor as it decreases activity of these interleukins. Despite these findings, large integrated safety datasets and analyses have not identified a coccidioidomycosis signal to date [8, 9].

### Diagnostic strategy and common pitfalls

Serologic testing is useful but may be blunted in immunosuppressed hosts; negative or low-titer results should not dissuade further work-up when imaging suggests peritoneal disease in an endemic-exposed patient [10]. Ascitic fluid cytology and culture are frequently non-diagnostic; diagnostic yield improves with tissue sampling. Our case suggests early diagnostic laparoscopy when feasible with multiple peritoneal biopsies to obtain histopathology and culture/polymerase chain reaction (PCR), particularly when imaging mimics carcinomatosis and fluids are negative [3, 10]. Histology demonstrating spherules within granulomas is highly specific. Culture remains a gold standard, and PCR (where available) may expedite confirmation. This approach avoids delays associated with serial fluid taps and allows earlier initiation of effective therapy.

### Therapeutic rationale and regimen selection

Infectious Disease Society of America (IDS) recommends antifungal therapy for all disseminated coccidioidomycosis cases; for stable non-central nervous system (CNS) disease, fluconazole 400 mg daily is first-line due to favorable pharmacokinetics, tissue penetration, and safety [1, 11]. Severe presentations, rapidly progressive disease, or extensive peritoneal involvement may warrant induction with liposomal amphotericin B followed by an oral azole agent [1, 11, 12]. In our patient, liposomal amphotericin B was chosen for rapid fungicidal effect given symptom burden and disease extent. Duration is typically at least 12 months and may be lifelong when immunosuppression persists or relapse risk is high [1]. For long-term suppression, fluconazole remains preferred; alternative azoles can be considered if intolerance or treatment failure occurs [11, 12].

### Prognosis, relapse, and follow-up

Mortality in disseminated coccidioidomycosis remains high, driven by host factors such as ongoing immunosuppression, diabetes, cirrhosis, disease burden, sepsis, and treatment delays [1, 2, 13]. Relapse is more likely when antifungal therapy is discontinued in the setting of persistent immunosuppression; prolonged or indefinite fungal suppression may be considered for patients who require ongoing biologic therapy [1]. Practical follow-up includes clinical monitoring, periodic imaging to document peritoneal response, and laboratory surveillance for azole safety. Coordination with rheumatology/dermatology is essential when discussing timing and necessity of biologic re-initiation.

### Prevention and pre-biologic screening

In endemic regions, Infectious Disease Society of America (IDSA) recommends pre-treatment coccidioidal serology before initiating biologic response modifiers, clinical vigilance during therapy, and holding the biologic if infection develops [1, 5]. International consensus statements do not mandate antifungal prophylaxis for IL-12/23 inhibitors, reflecting comparatively low serious infection risks versus tumor necrosis factor-alpha (TNF-α) inhibitors; nonetheless, individualized risk assessment is advised [14]. For ustekinumab candidates with prior coccidioidomycosis, shared decision-making about timing, prophylaxis, and monitoring strategy is prudent.

## Conclusion

Peritoneal coccidioidomycosis is an uncommon manifestation that can closely mimic carcinomatosis. In patients on ustekinumab living in or exposed to endemic regions, clinicians should maintain suspicion for Coccidioides when ascites and omental caking are present. Early diagnostic laparoscopy with biopsy, induction therapy for severe disease, and prolonged azole suppression are cornerstones of care. This case highlights a mechanistically plausible association between IL-12/23 blockade and disseminated disease that, although not reflected in safety databases, warrants clinical vigilance, and multidisciplinary coordination.
